# Masaoka-Koga and TNM Staging System in Thymic Epithelial Tumors: Prognostic Comparison and the Role of the Number of Involved Structures

**DOI:** 10.3390/cancers13215254

**Published:** 2021-10-20

**Authors:** Marco Chiappetta, Filippo Lococo, Luca Pogliani, Isabella Sperduti, Diomira Tabacco, Emilio Bria, Ettore D’Argento, Mariangela Massaccesi, Luca Boldrini, Elisa Meacci, Venanzio Porziella, Dania Nachira, Maria Teresa Congedo, Stefano Margaritora

**Affiliations:** 1 Università Cattolica del Sacro Cuore, Largo F. Vito 1, 00168 Rome, Italy; marco.chiappetta@policlinicogemelli.it (M.C.); luca.pojo88@gmail.com (L.P.); diomira_tabacco@yahoo.it (D.T.); emilio.bria@policlinicogemelli.it (E.B.); ettore.dargento@policlinicogemelli.it (E.D.); mariangela.massaccesi@policlinicogemelli.it (M.M.); luca.boldrini@policlinicogemelli.it (L.B.); elisa.meacci@policlinicogemelli.it (E.M.); venanzio.porziella@policlinicogemelli.it (V.P.); dania.nachira@policlinicogemelli.it (D.N.); mariateresa.congedo@policlinicogemelli.it (M.T.C.); stefano.margaritora@policlinicogemelli.it (S.M.); 2 Thoracic Surgery, Fondazione Policlinico Universitario A. Gemelli IRCCS, Largo F. Vito 1, 00168 Rome, Italy; 3 Biostatistics, IRCCS, Regina Elena National Cancer Institute-Rome, 00168 Rome, Italy; isabella.sperduti@ifo.gov.it; 4 Division of Oncology, Department of Medicine and Translational Surgery, Fondazione Policlinico Universitario A. Gemelli IRCCS, Largo F. Vito 1, 00168 Rome, Italy; 5 Department of Radiological, Radiotherapy and Hematology Sciences, Fondazione Policlinico Universitario A. Gemelli IRCCS, Largo F. Vito 1, 00168 Rome, Italy

**Keywords:** thymoma, thymic carcinoma, Masaoka–Koga, TNM, surgery

## Abstract

**Simple Summary:**

Thymic epithelial tumors were originally staged using the Masaoka–Koga staging system, even if recently the adoption of the tumor node metastases staging system was recommended. However, it remains controversial as to which staging system is the most effective in prognosis prediction for these patients. The aim of this study was to analyze the prognostic effectiveness of these staging systems and to verify a possible improvement.

**Abstract:**

Background: The aim of this study was to evaluate the Masaoka–Koga and the tumor node metastases (TNM) staging system in thymic epithelial tumors (TET) considering possible improvements. Methods: We reviewed the data of 379 patients who underwent surgical resection for TET from 1 January 1985 to 1 January 2018, collecting and classifying the pathological report according to the Masaoka–Koga and the TMN system. The number of involved organs was also considered as a possible prognostic factor and integrated in the two staging systems to verify its impact. Results: Considering the Masaoka–Koga system, 5- and 10-year overall survival (5–10YOS) was 96.4% and 88.9% in stage I, 95% and 89.5% in stage II and 85.4% and 72.8% in stage III (*p =* 0.01), with overlapping in stage I and stage II curves. Considering the TNM system, 5–10YOS was 95.5% and 88.8% in T1, 84.8% and 70.7% in T2 and 88% and 76.3% in T3 (*p =* 0.02), with overlapping T2–T3 curves. Including the number of involved structures, in Masaoka–Koga stage III, patients with singular involved organs had a 100% and 76.6% vs. 87.7% 5–10YOS, which was 76.6% in patients with multiple organ infiltration. Considering the TNM, T3 patients with singular involved structures presented a 5–10YOS of 100% vs. 62.5% and 37.5% in patients with multiple organ involvement (*p =* 0.07). Conclusion: The two staging systems present limitations due to overlapping curves in early Masaoka–Koga stages and in advanced T stages for TNM. The addition of the number of involved organs seems to be a promising factor for the prognosis stratification in these patients.

## 1. Introduction

Thymic epithelial tumors (TET) are rare tumors occurring in the anterior mediastinum, and surgery is the treatment of choice ensuring excellent results in terms of disease control and long-term survival [1,2].

Tumor staging started in the 80s, with a classification proposed by Dr. Masaoka, which was then revised with Dr. Koga (Masaoka–Koga staging system) considering 4 stages and classifying TET based on the infiltration of the neighboring structures or lymphatic/hematogenous spreading [3]. In recent years, the international association for the study of lung cancer and the international thymic malignancy interest group [4] proposed a different classification for TET, based on the indication of the Union for International Cancer Control (UICC) and the American Joint Committee on Cancer (AJCC).

Despite some concepts being similar to the previous staging system, in the proposed TET tumor node metastases (TNM) staging system the nodal factor was revised. The tumor factor was revised, including tumors with pleural invasion as T1 underlining the different prognosis in patients with pericardial infiltration re-classifying them as T2 and leaving a category of patients with other infiltrated structures, such as T3 [5]. One of the most important challenges in TET staging is that the involvement can include one or more structures and the involvement of some structures may imply an involvement of another, e.g., lung infiltration is only possible after mediastinal pleural infiltration. For these reasons, the TNM is based on the level of the infiltration concept, including the tumor in a certain “level” of involvement if either one or more than one structure of that level is involved, with or without the explicit involvement of structures included at a lower level [5]. However, few studies compared the two staging systems [6,7,8,9], reporting controversial results in terms of prognosis, especially comparing advanced stages or considering overall survival, while a better performance was demonstrated considering stage I or disease-free survival (DFS). Another interesting point regards the role of the number of the involved structures, not considered in actually available staging systems and in validation studies, which may be beneficial for a better prognosis stratification. 

The aim of this study was to compare the tumor factor of the Masaoka–Koga and the TNM staging systems, while also investigating the potential role of the number of involved structures.

## 2. Materials and Methods

### 2.1. Clinical and Pathological Records of Patients who Underwent Surgical Treatment for TET in our Institution from 1 January 1985 to 1 January 2018 Were Collected and Retrospectively Reviewed 

Preoperatively, every patient underwent neurological evaluation for myasthenia gravis (MG), completing the diagnostic item with anti-acetylcholine antibody receptors and electromyography if needed. 

Surgery was indicated in the case of suspected TET, demonstrated with thorax computed tomography with contrast, and patients were judged able to tolerate TET resection in general anesthesia. In case of doubt, magnetic resonance and fine needle biopsy was performed to determine infiltration of neighboring structures or to obtain a preoperative diagnosis. 

From 1985 to 2014, surgery was performed via median sternotomy, while from 2014, robotic-assisted thymectomy was performed in selected cases with a tumor diameter less than 3 cm and without infiltrated neighboring structures. All patients underwent extended thymectomy, including the peri-thymic fat and the en-bloc, with the neighboring structures in the case of suspected infiltration. All surgeries were performed with the aim to obtain a radical resection, and in the case of macroscopically residual of disease or doubt of microscopically residual of disease, the area was marked with titanium clips to permit identification for further adjuvant radiotherapy. 

Pathological specimens were staged according to the Masaoka–Koga staging system [3], while since 2014, a referral to the TNM staging system has also been reported. Regarding patients operated before the TNM adoption, the pathological report has been reviewed and re-staged according to the TNM. Histology was categorized in accordance with the WHO [10]. 

The number of involved structures were counted, reconsidering the pathological report and adding the infiltrated structures by the different TET foci, e.g., in case of different tumor foci invading the anonymous vein on the top and the pericardium inferiorly, the count was two structures. The count always included structures infiltrated before the last one reported if the anatomy implies its infiltration, e.g., in the case of lung infiltration, the mediastinal pleura must be crossed by the tumor before infiltrating the lung, so the number of involved structure results in 2 (mediastinal pleura + lung). If another structure, such as an anonymous vein or pericardium involvement was present, the count was 3. 

Induction therapy was indicated considering pre-operative imaging and after multidisciplinary discussion, while adjuvant therapy (AD) was indicated based on pathological results. In particular, medical oncologist or the radiotherapist decided on chemotherapy (CT) and/or radiotherapy (RT) based on tumor characteristics, patient clinical conditions and previous treatment incidences. CT and RT regimens changed alongside the study period, always following the most recent guidelines available, while follow-up was conducted by clinical evaluation and thoracic-computed tomography every year for 5 years for stage I/II thymomas. For R1–R2 resections or stage III/IV thymomas and thymic carcinoma follow-up consisted of a CT scan every 6 months for 2 years, then annually for 10–15 years [11,12]. 

Patients affected by MG also underwent neurological surveillance (clinical evaluation and lab tests), and a computed tomography scan was anticipated in the case of clinical worsening of symptoms.

### 2.2. Statistical Analysis

A descriptive analysis, including clinical and demographic characteristics of patients, was performed, analyzing the median and range for continuous variables and the absolute value and relative frequencies for categorical variables. Overall survival (OS) was calculated from the date of surgery to death for any cause. DFS was calculated from the time of surgery to the first detection of recurrence. Survival curves were calculated by the Kaplan–Meier product-limit method from the date of surgery until relapse or death. The log-rank test was used to assess differences between subgroups. The hazard ratio (HR) and the 95% confidence intervals (95% CI) were estimated using the Cox univariate model. Significance was defined at the *p* ≤ 0.05 level. Statistical analyses were performed by use of SPSS (v. 21.0, SPSS Inc., Chicago, IL, USA).

## 3. Results

### 3.1. Overall

Clinical and pathological characteristics are reported in Table 1. In detail, the major part of patients presented early stages and received a complete resection. The pleura and the pericardium resulted as the most common infiltrated structures and multiple involvement was present in 37 (12.4%) patients. Myasthenia gravis was present in the 75.5% of cases. 

Five- and ten-year overall survival (5–10YOS) was 94.2% and 86.4%, while five- and ten-year disease-free survival (5–10YDFS) was 87.8% and 77.9%. Mean follow-up was 146 months (range 1–577).

10YDFS resulted in 80%, 79%,78.5% and 70.9% in patients with none, 1, 2 and 3 or more involved structures, respectively (*p =* 0.28), while 10YOS resulted in 87.8%, 87.5%, 80% and 76.4% in patients with none, 1, 2 and 3 or more involved structures, respectively (*p =* 0.16).

### 3.2. Masaoka–Koga Staging System

The majority of patients presented early stage tumors (Table 2), and only three cases resulted in being Masaoka–Koga stage 4 for pleural-pericardial dissemination. No difference in terms of the number of infiltrated organs were present, considering histology and kind of resection. In particular, the number of infiltrated organs were 0 in 80.7%, 1 in 7.4%, 2 in 8.8% and 33 or more in the 3% of patients with thymoma vs. 0 in 66.7%, 1 in 8.3%, 2 in 16.7% and 3 or more in the 8.3% of patients with thymic carcinoma (*p =* 0.55); considering the kind of resection, the number of infiltrated organs were 0 in 80.3%, 1 in 7.4%, 2 in 9% and 33 or more in the 3.2% of patients with complete resection vs. 0 in 66.7% and 2 in 33.3% of patients with incomplete resection (*p =* 0.51).

**Figure 1:**
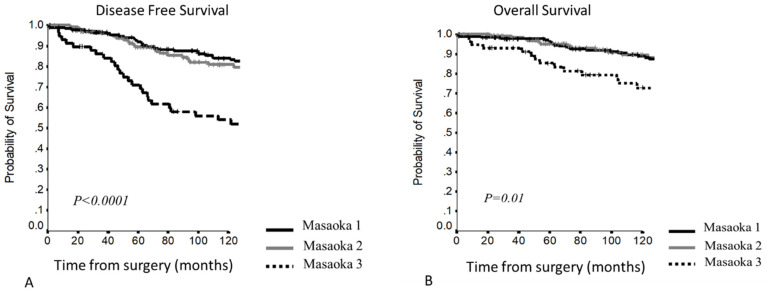
(**A,B**) Disease-free survival and overall survival according to the Masaoka–Koga staging system.

Survival outcome resulted as follows:

5–10YDFS (*p* < 0.0001) (Figure 1A)
  Stage I: 92.1% and 84%
  Stage II: 89.5% and 80.8%
  Stage III: 71% and 54%
5–10YOS (*p =* 0.01) (Figure 1B)
  Stage I: 96.4% and 88.9%
  Stage II: 95% and 89.5%
  Stage III: 85.4% and 72.8%

Considering the different stages, stage I and II curves overlapped, while a clear stratification for survival was present comparing stage III (Figure 1). At Cox regression analysis, significant differences were confirmed for all stage comparisons, except for stage I vs. stage II (Table 3).

**Figure 2:**
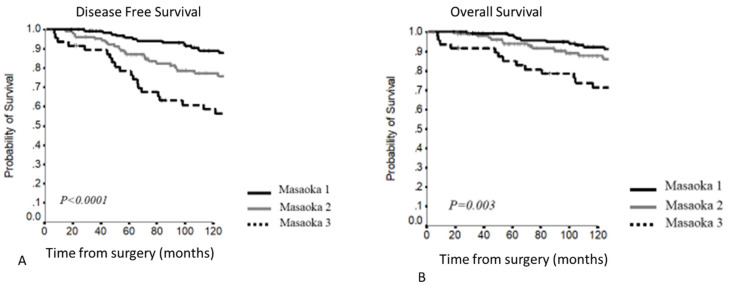
(**A,B**) Disease-free survival and overall survival according to the Masaoka–Koga staging system in MG patients.

This staging system was effective to predict DFS (*p* = 0.001) and OS (*p* = 0.03) and also in selecting myasthenic patients (Figure 2), but considering the DFS in myasthenic patients, stage I and II curves presented a clear separation: stage I 5–10YDFS 95.8% and 88.8% vs. stage II 5–10YDFS 87.2% and 77.3%. 

**Figure 3:**
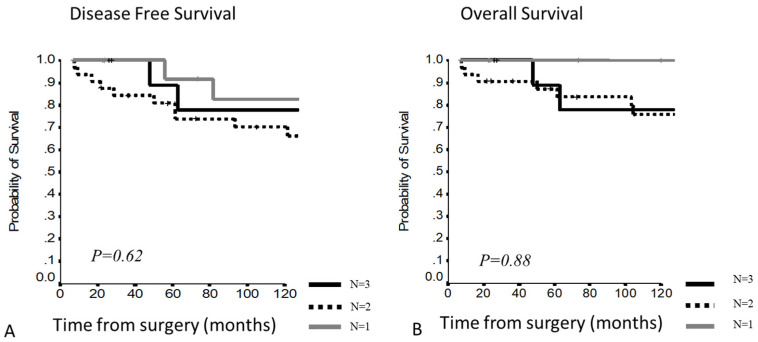
(**A,B**) Disease-free survival and overall survival according to the number of involved structures in Masaoka–Koga stage 3.

In stage III, no significant differences were present considering the number of involved structures: 10Y-DFS of 82.5%, 77.8% and 70.2%; 10Y-OS of 100%,77.8% and 75.9% in the case of 1, 2 or 3 or more involved structures, respectively (Figure 3). 

**Figure 4:**
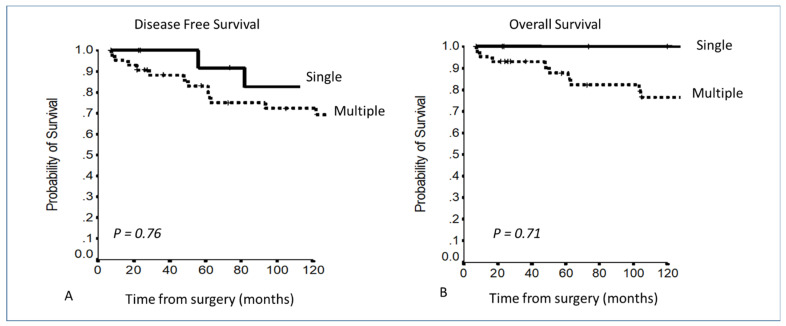
(**A,B**) Disease-free survival and overall survival in Masaoka–Koga stage III according to the number of involved structures.

Comparing patients with a single involved structure vs. patients with more than 1 involved structure, a clear curve separation was present, even if it was not statistically significant: 5–10YDFS 91.7% and 82.5% vs. 83% and 72.2% (*p* = 0.76); 5–10YOS 100% vs. 87.7% and 76.6% (*p* = 0.71) (Figure 4).

### 3.3. TNM Staging System 

Considering the TNM, 25 patients presented pericardial infiltration and were categorized as T2, while 32 patients were staged as T3, presenting a multiple structure infiltration in 28 cases (Table 4).

Survival outcome resulted as follows:

5–10YDFS (*p* < 0.0001)
  T1: 90.13% and 82.1%
  T2: 76% and 56.0%
  T3: 71.6% and 56.5%
5–10YOS (*p =* 0.02)
  T1: 95.5% and 88.8%
  T2: 88% and 70.7%
  T3: 84.8% and 76.3%

**Figure 5:**
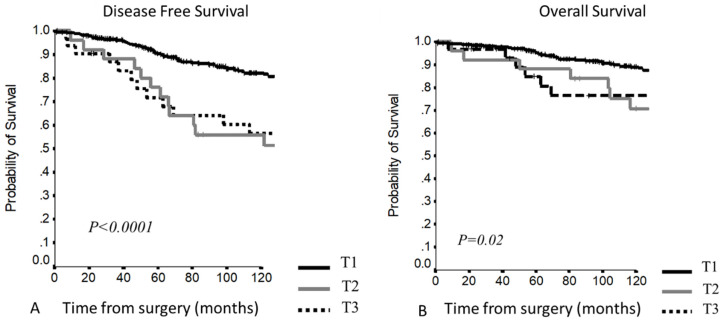
(**A,B**) Disease-free survival and overall survival according to the TNM staging system.

This staging system presented a clear separation between T1 and T2–T3 curves, but the last two curves overlapped and patients with pericardial infiltration showed a worse survival compared to patients with other involved structures (Figure 5). 

At Cox regression analysis (Table 4), a significant difference in DFS was present comparing T1 vs. T2 and T3, while no significant differences were present comparing T2 vs. T3 patients. Regarding OS, a significant difference was present comparing T1 vs. T3, while comparing T1 and T2, the difference was not significant, even if the HR was 1.699.

**Figure 6:**
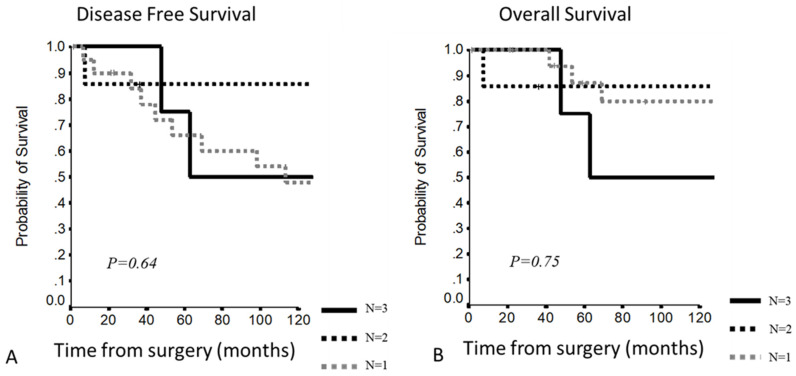
(**A,B**) Disease-free survival and overall survival according to the number of involved structures in T3 patients.

In T3 patients, the 10YDFS resulted in 48%, 85.7% and 50% in patients with 1, 2 and 3 or more involved structures (*p =* 0.64); 10YOS resulted in 79.8%, 85.7%, and 50% in patients with 1, 2 and 3 or more involved structures, respectively (*p =* 0.75) (Figure 6).

**Figure 7:**
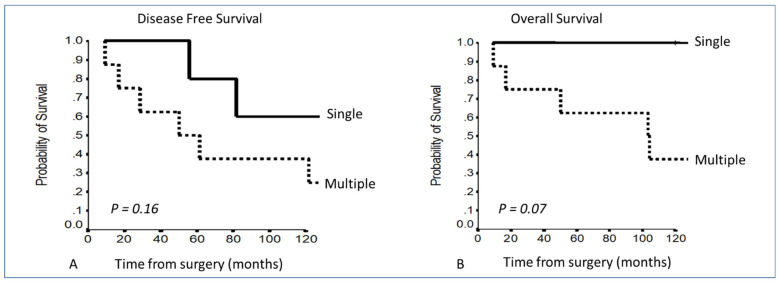
(**A,B**) Disease-free survival and overall survival in T3 of the TNM according to the number of involved structures.

Considering the number of involved structures, patients with a single involved structure presented a benefit in survival, which was not significant considering the DFS, raising the significance considering OS: 5–10YOS 100% in a single involved structure vs. 62.5% and 37.5% in patients with multiple involved structures (*p* = 0.07) (Figure 7).

**Figure 8:**
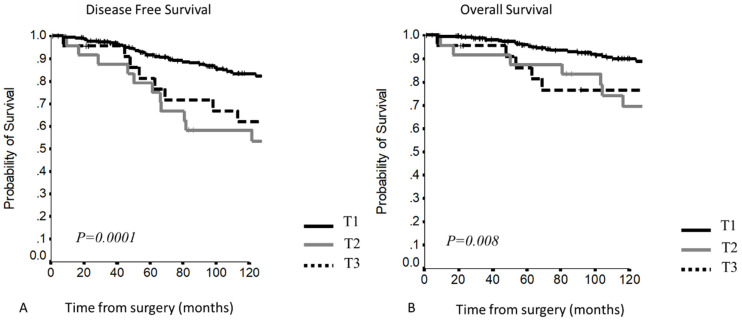
(**A,B**) Disease-free survival and overall survival according to the TNM staging system in MG patients.

Finally, considering myasthenic patients, the T classification resulted in being effective for prognosis prediction, even if T2–T3 curves overlapped, while the presence of pericardial infiltration seems to be associated with a worse prognosis compared to T3 patients (Figure 8).

## 4. Discussion

In our study, we validated the two staging systems present for TET in a large single-center cohort, pointing out the limitations and advantages. In particular, analyzing the survival outcome according to the Masaoka–Koga staging system, a stage separation considering the tumor extracapsular invasion without neighboring structures invasion seems to be redundant. Indeed, any survival differences were present in a comparison of stage I and stage II, with overlapping survival curves, while a clear survival difference was present comparing stage I–II vs. stage III. Our results are in agreement with a few other validation studies [6,7] that showed, if present, significant OS differences comparing stage I–II vs. III or reported significant differences considering DFS only. Conversely, we noted a good stage separation considering the T factor in the TNM staging system, with a significant difference comparing T1,T2 and T3 regarding DFS, while the difference was not significant for OS, even if the HR for T2 patients was 1.677 when compared with T1. The effectiveness of the TNM for DFS prediction was pointed out by Fukuia et al. [7], reporting a good performance for DFS prediction adopting this staging system. 

On the other hand, no survival differences were detected comparing pericardial vs. other infiltrated structures. These results are in line with Liang et al. [6], who showed any survival difference comparing T2 and T3 patients. These results confirmed that the T factor proposal [5] did not result in differences regarding OS, even if T3 patients presented a significantly high recurrence rate. 

The T factor categorization remains the most important limitation of the IASCL/ITMIG proposal due to contrasting results reported in other studies [5,6], including in our research. For this reason, we performed a sub-analysis considering the number of involved structures, a parameter not sufficiently investigated in previous literature, which may explain possible survival differences. 

Analyzing this variable in Masaoka–Koga stage 3, which includes all patients with structure infiltration, a survival curve separation was present, with 100% of long term survival in cases of single structure infiltration, even if this was not statistically significant. This result should be carefully considered and needs a larger number of patients to be verified. 

Similarly, we performed the same analysis on T3 patients, and even in this group, we found a separated survival curve for DFS and an increased statistical significance analyzing OS (*p* = 0.07). Our analysis was performed in a limited, but not negligible, number of patients, and even if our results are encouraging, further studies with a large database are needed to validate our findings. Indeed, it is possible that the number of infiltrated structures may be used for future analysis in order to improve survival stratification in these patients. 

However, the T factor was fundamental in TET management and different components, such as pleural and pericardial infiltration, may require a specific T category [13]. For these reasons, specific analysis of the local evolution of TET may be taken into account, investigating not only the level of infiltration, but also possible different involved structures.

Indeed, despite this, the TNM proposal, based on the concept of infiltration level, includes two separate categories for pericardial (T2) or other infiltrated structures (T3), and this anatomical difference did not reflect a significant OS difference [5]. This model, valid for DFS prediction, may present limitations: tumor growth is not considered, and the pericardium may not be the first involved structure or a different area of infiltration may be presented. For example, a tumor growing in the upper region may directly infiltrate the anonymous vein without involving the pericardium. In our series, only 4 out of 11 patients with vascular infiltration, and only 8 out of 26 patients with lung infiltration. presented with concomitant pericardial infiltration, confirming this hypothesis. On the other hand, only 37% of patients with an infiltrative component presented a single structure infiltration, suggesting that this classification also may be considered for further staging systems. 

Another possible solution is to use models that include the T proposal of the TNM staging system and histology, which is a solution that might be effective also considering the OS prediction [14,15]. However, we agree with the IASLC/ITMIG staging committee, postulating that a staging system should be adopted for all histology types [4], and the scientific community can focus on this kind of staging system. On the other hand, composite models in TET management may be used in the setting of integrated treatments. Indeed, the role of induction/adjuvant therapy in TET remains debated, with encouraging results, especially in the last few decades [16,17]. However, the presence of an appropriate staging system may be extremely useful to realize post-operative treatment guidelines.

Leuzzi et al. [16] reported a significant OS improvement in the case of adjuvant therapy administration in patients with infiltrative thymomas. The sub-analysis confirmed the survival benefit for T3—but not for T2—patients. Despite this, a limited number of T2 patients may explain the lack of statistical significance. This study explains the importance of appropriate staging to manage these patients. 

Starting from these considerations, the inclusion of the number of involved structures may be useful from different points of view. First, the introduction of a classification system, with a different concept for stage definition, may reduce confusion, simplifying its adoption by physicians. Indeed, in a recent survey, 78% of members of the major thymic organizations worldwide consider the TNM useful, despite the Masaoka–Koga system still needing to be adopted by 87% [13]. 

Despite the TN, which especially changed the N and M parameters, the T factor presents similar categories compared with the Masaoka–Koga, and this might reduce the transition to a different staging system. On the other hand, proposing a different T classification may accelerate this change. 

Another point regards the possibility of better patient management in terms of follow-up schedules and integrated treatments. Considering that the number of involved structures may give different information regarding the tumor direction, spreading and infiltrating foci permit modulated treatments based on tumor and patient characteristics. However, further studies with a higher number of patients are needed to really test the outcome after adjuvant therapy, according to the number of involved structures. 

Moreover, analyzing the time of recurrence based on the number of involved structures, follow-up schedules and indications could be potentially modified, which are currently based on histology and the presence of infiltration [11,12]. 

Finally, we analyzed survival in myasthenic patients that presented a similar survival trend, confirming the advantages and limitations of the Masaoka–Koga and TNM staging systems. However, a clear curve separation was present for DFS using the Masaoka–Koga staging system. MG symptom recurrence or deterioration may anticipate recurrence diagnosis between the different stages, explaining our curve separation results. We are aware that our population presented a particularly high percentage of myasthenic patients, because our center is a national landmark for MG diagnosis and treatment. On the other hand, this elevated number of MG patients permitted an ad hoc analysis. 

This study presents some limitations. First, due to its retrospective nature, it is hard to reconstruct the pre-operative work-up or the post-operative follow-up for each patient. On the other hand, the single center design permits us to assume a homogenous management. Second, in the long study period, different adjuvant therapies or surgical approaches were adopted, but adjuvant treatment was administered according to the available guidelines and surgery was performed with the aim of performing a complete and radical thymectomy, independent of the surgical access.

## 5. Conclusions

Our study confirms the effectiveness of the Masaoka–Koga and TNM staging system for survival prediction regarding the tumor parameter in TET, underling the potential limitations regarding the I–II stage definition in the Masaoka–Koga and T2–T3 definitions in the TNM staging system.

The adoption of other parameters, such as the number of involved structures, seems encouraging to improve these systems, but requires a further evaluation in larger patient cohorts.

## Figures and Tables

**Table 1:** Clinical and pathological characteristics.

	N. of Patients
Age (mean and SD)	51.2 ± 14.65
Gender	
Male	176 (46.4%)
Female	203 (53.6%)
Comorbidity	
Myasthenia Gravis	286 (75.5%)
Diabetes	39 (10.3%)
Basedow’s disease	3 (0.8%)
Hashimoto’s thyroiditis	60 (15.8%)
Pure red cell aplasia	18 (4.7%)
Other autoimmune disorders	21 (5.5%)
Surgical Radicality	
R0	375 (98.9%)
R1	3 (0.8%)
R2	1 (0.3%)
Involved Structures	
Pleura	60 (15.8%)
Pericardium	37 (9.8%)
Lung	26 (6.9%)
Great Vessels	11 (2.9%)
N. of infiltrated organs per patients	
0	304 (80.2%)
1	28 (7.4%)
2	35 (9.2%)
3	12 (3.2%)
Histology	
A-AB	71 (18.8%)
B1	51 (13.4%)
B2	194 (51.2%)
B3	50 (13.2%)
C	13 (3.4%)

**Table 2:** Pathological stages according to the Masaoka–Koga staging system.

Masaoka–Koga		
Stage	Patients	Rate
I	179	47.2%
II	140	36.9%
IIa	49	12.9%
IIb	91	24%
III	57	15.1%
IV	3	0.8%

**Table 3:** Cox regression analysis for disease-free survival and overall survival according to the Masaoka–Koga and TNM staging system.

Stage	Disease Free Survival		Overall Survival	
	HR	*p* Value	HR	*p* Value
Masaoka–Koga				
		<0.001		0.013
II vs. I	1.050	0.831	0.979	0.936
III vs. I	2.701	<0.001	2.027	0.006
II vs. III	0.389	<0.001	0.483	0.015
T TNM				
		<0.001		0.019
2 vs. 1	1.909	0.025	1.699	0.103
3 vs. 1	3.167	<0.001	2.267	0.012
2 vs. 3	0.603	0.162	0.750	0.502

**Table 4:** Patient distribution according to the TNM staging system.

TNM		
T	Patients	Rate
1a	304	80.2%
1b	15	4%
2	25	6.6%
3	32	8.4%
4	3	0.8%

## Data Availability

The data are the property of the Institution and cannot be shared.
